# Research and Development for Botanical Products in Medicinals and Food Supplements Market

**DOI:** 10.1155/2013/649720

**Published:** 2013-03-28

**Authors:** Marco Miroddi, Carmen Mannucci, Ferdinando Mancari, Michele Navarra, Gioacchino Calapai

**Affiliations:** ^1^Department of Clinical and Experimental Medicine, University of Messina, 98125 Messina, Italy; ^2^Pharmaco-Biological Department, Faculty of Pharmacy, University of Messina, 98168 Messina, Italy; ^3^Operative Unit of Clinical Pharmacology, Azienda Ospedaliera Universitaria Policlinico “G. Martino”, 98125 Messina, Italy

## Abstract

Botanical products sold in the health area are generally intended as drugs, medicinal products, food supplements or substances for therapeutic use. Use of botanicals for improving or to care human health has evolved independently in different countries worldwide. Regulatory issues regarding botanical products designed for the food supplements or medicinal market and their influence on research and development are discussed. European Union (EU) and United States (US) policies regulating these products are focused with comments on the legislations delivered during the last ten years and differences existing in rules between these countries are emphasized. Research and development on botanical products nowdays strongly influenced by the product destination in the market. Addressed and differentiated research for either food supplements or medicinal markets is necessary to purchase data really useful for assessment of safe and effective use for both the categories. The main objective is to catalyze interest of academic and companies' researchers on crucial aspects to be taken into account in the research for the development of botanical products.

## 1. Introduction

In the scientific area, botanical products are generally intended as drugs, medicinal products, food supplements, or substances for therapeutic use derived from raw material of whole plants or parts of them. Starting from these materials, botanical substances (e.g., whole, fragmented or cut plants, algae, fungi, lichens) or botanical preparations are obtained through various processes such as extraction, distillation, purification, concentration, fermentation, and others. In many countries these products are regulated both as medicinal products and as food supplements and they are often labeled as natural foods or sport supplements [1]. 

Botanical products are widely available to consumers through different distribution channels. In particular, they are sold over the counter in pharmacies and can be bought also in supermarkets, herbalist's shops, or through Internet [2]. Use of botanicals to improve or to care human health has evolved independently in the world, depending on specific cultures, current medical and nutritional practices, availability of botanical species and main policies of established companies on the territory. In consequence of this, there are different ways in which countries define medicinal plants or herbs or botanical products derived from them, and countries have adopted various approaches to licensing, dispensing, manufacturing, and trading to ensure their safety, quality, and efficacy, and due to these reasons herbal preparations vary from country to country [3]. Moreover, national legislations have also facilitated and addressed directly or indirectly the marketing of traditional botanical products as food supplements or as medicinal products [4].

For the present paper, we chose to describe shortly the most important regulatory issues being in force in EU and US, two of the largest markets for botanicals, and to highlight the most important steps to follow in the modern research and development of health botanical products. 

## 2. Botanicals in Herbal Medicinal Products Market

European Union (EU) policy regulating botanical products is illustrated in different legislations delivered during the last ten years. After decades of almost exclusive commercialization of herbal with medicinal properties through food supplements market, an effort to harmonize medicinal use of herbal substances through draw and delivery of the so-called Traditional Herbal Medicinal Product Directive 24/EC/2004 (THMPD) has been made. THMPD came into force in April 2011 and aimed to protect public health and at the same time secure the free movement of herbal products within the EU. This directive is the legal basis of regulation for the use of herbal medicines in phytotherapy in European countries. Once implemented, the objective of the directive is to remove in the EU the constraints that have made it difficult to grant marketing authorizations of herbal substances and preparations as traditional medicinal products under the pre-existing communitary legislation [5]. 

The Directive 2004/24/EC established that “*herbal medicines are any medicinal product exclusively containing, as active ingredients, one or more herbal substance, one or more herbal preparation or more such herbal substances in combination with one or more such herbal preparations*.” The greatest novelty contained within the directive is the amendment of the previous 2001/83/EC [6], by establishing that herbal medicinal products release in the market needs authorization as well as required for drugs. In other words, according to the 2004/24/EC, it is mandatory that botanicals authorized as medicinal products, before commercialization, have to undergo an evaluation procedure following the submission of an application for simplified registration [5]. 

The 2004/24/EC establishes that in European countries, for registration of botanicals as medicinal products, companies refer to one unique set of information on a herbal substance or herbal preparation purchased through the community monograph drawn by an “ad hoc” committee. The Herbal Medicinal Products Committee (HMPC) has been established at the European Medicines Agency (EMA, London), the agency which is responsible for the scientific evaluation of medicines developed by pharmaceutical companies for use in the European Union. Community herbal monographs comprise the scientific opinion of the HMPC on safety and efficacy data concerning a herbal substance and information on what are adequate herbal preparations intended for medicinal use. For any single plant, each herbal preparation is assessed individually according to the available information and it may vary from one preparation to another. The set of information included in the monograph comprises clinical (indication, posology, etc.) and safety (warnings, recommendations, and contraindications) issues [7]. 

The Directive 2004/24/EC introduced two subcategories of herbal products that can be commercialized as medicinal products. One of the subcategories of herbal products is that for which is possible to collect clinical data showing efficacy for the intended use, and data demonstrating a positive risk/benefit profile. These products have been called herbal medicinal products with a “well-established use” (WEU) on the basis of the existence of body of evidence sufficient to show clinical efficacy in specific therapeutic indications. The second subcategory, comprising products derived from more numerous plants than WEU products, is represented by “traditional herbal medicinal products” (THMPs). THMPs are called botanical products for which proof of clinical efficacy is not existing, or poor or not sufficiently convincing. The Directive establishes that this subcategory can be registered as traditional medicinal products, only if the use in specific therapeutic indications (not requiring medical supervision) is plausible according to their traditional long standing utilization and if they present an acceptable safety level. Moreover, products are eligible for license as a traditional herbal medicine only if they have been used to treat a specified health complaint for at least 30 years, including a minimum of 15 years at least in one country of EU. Obviously, WEU and THMPs are held to satisfy similar safety and quality standards as pharmaceutical drugs [8].

For THMPs authorization, the 2004/24/EC contemplates a simplified registration after that application is presented to national agencies regulating drug market. Finalized (definitive) versions of community monographs have to be taken into account by the member states when examining an application for herbal medicinal products. Even though the member states are not obliged to follow the monographs, any decision not to accept the content of the monograph as it is adopted by the HMPC should be duly justified [3, 9]. HMPC has defined that at least one controlled clinical study or alternatively a well-documented clinical experience with sufficient supportive pharmacological data is needed to substantiate efficacy for a well-established use [10]. The current situation regarding the status of products registration is that in some European member states, herbal preparations have been regulated under food law, although they have pharmacological properties, and the tendency in these countries remains to place typical medicinal plants on the market as food products [8].

A “community list of herbal substances, preparations, and combinations thereof for use in traditional herbal medicinal products” has been established in Europe. It is based on proposals from HMPC and is gradually developed. When herbal substances/preparations are included in the Community list, registrations for traditional herbal medicinal products containing them have a significant advantage. The reason for this advantage is that once a traditional product is based on a herbal substance/preparation included in the Community list, the applicant will not be required to provide evidence of the safe and traditional use for its registration if the intended use and related claims in the application comply with the information contained in the list. Moreover, national competent authorities will not have the opportunity to require additional data to assess the safety and the traditional use of the product [5]. Community monographs are published by HMPC whereas list entries are published by the European Commission and have therefore a broader legal status. List entries are legally binding and competent authorities will not request additional data to assess the safety and traditional use of the product [10].

In the United States, categorization of botanicals is based on intended use, safety, regulatory status, and degree of characterization [11]. In the field of medicinals, products can be “prescription drugs” or “over-the-counter drugs.” Authorization for this category of products requires rigorous testing including three distinct phases of clinical testing to ensure safety and efficacy and close scrutiny by the Food and Drug Administration (FDA). Today in United States, about 25% of the drugs used are based on plant-derived products [12]; however, only pure compounds isolated from plants and subjected to the same rigors as synthetic pharmaceutical can be conventional drugs [13].

The guidelines for registration of botanical drugs were released in 2004. Botanical drugs are evaluated for safety and clinical efficacy just as conventional drugs, but the process for botanical drugs can be accelerated on the basis of the empiric knowledge of safety derived from observation in human use. Botanical drugs are produced under the same strictly regulated quality conditions as conventional pharmaceuticals [11, 14].

## 3. Botanicals in Food Supplements Market 

Use of plant products as supplements for food originates from a long tradition where the consumption of herbal infusions, digestives, juices, elixirs, and extracts had the purpose to maintain and promote health [15]. In Europe, Regulation (EC) No. 178/2002 defines “food” (or “foodstuff”) as “*any substance or product, whether processed, partially processed or unprocessed, intended to be, or reasonably expected to be ingested by humans.*” Definition of “food” includes drink, chewing gum and any substance, water included [16]. Botanical products represent a principal ingredient, alone or in association with other substances, of food (or dietary) supplements. The food use as supplements has been ruled by the Food Supplements Directive (FSD) 2002/46/EC that established the definition of food supplements as “*foodstuffs the purpose of which is to supplement the normal diet and which are concentrated sources of nutrients or other substances with a nutritional or physiological effect …” *[17]. With this definition is expressed the concept that food supplements have no therapeutic function, whereas health-keeping functions are emphasized. 

Successively, food supplements context was further regulated through the delivery of the “*Regulation n. 1924/2006 of the European Parliament and of the Council released on 20 December 2006 on nutrition and health claims made on foods*.” It establishes the concept of “claim,” intended as “*any message or representation which states, suggests or implies that a food has particular health characteristics*.” In the same Regulation, “health claim” is defined as “*any claim that states, suggests, or implies that a relationship exists between a food category, a food or one of its constituents and health*”. Aim of the Regulation is ruling the issue regarding intended health indications for food use through the “health claims”. The Regulation 1924/2006 establishes that “nutrition claims” (for products with nutritional properties) and “health claims” may be used in the labeling, presentation, and advertising of foods placed on the market in the EU only if they comply with the defined provisions. The use of a health claim requires an authorization by the European Commission through the Standing Committee for Food Safety and Animal Health and a scientific assessment by European Food Safety Authority (EFSA) to ensure that they are based on “*generally-accepted scientific evidence, taking into account the totality of the available scientific data, and by weighing the evidence*” [12].

On the basis of exclusive physiological function of food, the new policy points out that claims have to recall the health-keeping and nontherapeutic role of food supplements. The same regulation establishes that claims are also accepted in the case of “Reduction of disease risk claims” intended as “*any health claim that states, suggests or implies that the consumption of a food category, a food or one of its constituents significantly reduces a risk factor in the development of a human disease*”. To obtain authorization for a claim, companies have to produce an application to the Member States, which will submit it to the EFSA. 

Whether or not, available data for each claim are sufficient to substantiate the claim (on the basis of accepted scientific evidence) is a scientific judgement of EFSA. This decision on the use of the claim will be taken by EFSA, after an accurate examination of the relevant scientific literature corroborating the requested claim [4].

Health claims for botanical products have become a crucial issue, since authorization of the great part of claims proposed for botanical products have been refused by EFSA. Following this decision, a criticism has been raised from companies, asking and suggesting for a more tolerating regulatory approach. In particular, it has been objected that it may be more difficult for consumers to fully understand, in the absence of health claims, the benefits, if any associated with the consumption of the product. Another objection is the idea that the current regulatory and legal situation in the EU is not adequate to lead to a harmonization that would make it possible for all European citizens to benefit from traditional botanical food supplements and medicinal products under, if not identical, at least comparable conditions [4].

Differences in botanicals regulation between Europe and United States are existing. Food supplements were previously defined in United States by the Dietary Supplement Health and Education Act (DSHEA) released in 1994 as products taken by mouth that contain a “dietary ingredient” intended to supplement the diet. DSHEA changed the marketing and legal climate for dietary supplements and herbs and enabled the exponential growth of product sales since that time. This Act amended previous statutes to encompass dietary supplement-specific provisions, including the definition of dietary supplements, product safety, nutritional statements and claims, ingredient and nutritional labeling, good manufacturing procedures, and the classification of “new” dietary ingredients. As defined by DSHEA, a dietary supplement is a product other than tobacco that is intended to supplement the diet and contains one of the following dietary ingredients: a vitamin, a mineral, an herb or other botanical, an amino acid, a dietary substance to supplement the diet by increasing the total daily intake, or a concentrate, metabolite, constituent, extract, or combinations of these ingredients [18]. 

The Nutrition Labeling and Education Act of 1990 gives the United States FDA the authority to regulate health claims on food labels [19]. These claims describe the link between specific nutrients or substances in food and a particular disease or health-related condition [20]. The “Nutrition Labeling and Education Act” permits use of health claims if there is evidence to support the claim and significant scientific agreement among qualified experts about the claim, and if the claim is not misleading. The FDA Modernization Act of 1997 permits manufacturers to use health claims based on authoritative statements by a scientific body of the US government, such as the National Institutes of Health [21]. Three categories of claims can currently be used on food and dietary supplement labels in the US: (1) health claims, (2) nutrient content claims, and (3) structure/function claims. Structure/function claims were authorized under the Dietary Supplement Health and Education Act of 1994 and describe the effect of a dietary supplement on the structure or function of the body [14]. In US, health claims are authorized by FDA only after a systematic review of scientific evidence [22]. Only studies conducted in “healthy populations” are considered, because health claims are directed to the general population or designated subgroups (e.g., elderly persons) and are intended to assist the consumer in maintaining healthful dietary practices. Health claims are limited to claims about risk reduction and cannot be about the diagnosis, cure, mitigation, or treatment of disease. The FDA exerts its oversight in determining, by means of the following Acts, which nutrient content claims may be used on a label or in labeling: (a) the Nutrition Labeling and Education Act (NLEA) of 1990, by issuing a regulation authorizing a nutrient content claim, and (b) the FDA Modernization Act of 1997, by prohibiting or modifying by regulation a nutrient content claim. The NLEA required that the FDA issue regulations for authorizing the use of a health claim about a substance/disease relationship only when the significant scientific agreement standard was met [23]. 

In United States, companies are responsible for the safety of their products and food supplements, including those containing botanicals, which do not need approval from Food and Drug Administration (FDA) before commercialization. Only in the case of new ingredients market introduction, legislation requires a report including safety but not efficacy data. In conclusion, botanical products are generally sold in United States as food supplements with no particular authorization needed for their release in the market, while companies have to show the truthfulness of claims [22]. 

## 4. Research and Development for Botanical Products

Until about two decades ago, scientific investigation on medicinal plants has been characterized for the most part by *in vitro* or *in vivo* scientific evidence. Most of all, they were preclinical demonstrations of one or more biological and pharmacological activities of extracts or other herbal preparations obtained from the whole plant or parts of the plant. According to which is indicated by legislations, it is becoming clear that research supporting the use for health of botanical products has to be addressed in line with final destination in the market. Between food supplements and medicinals markets, the fundamental difference is represented by the “intended use” of each category of products. This is “health-keeping” for food supplements and “therapeutic” for medicinal products. New legislations have established that authorization needs scientific demonstration either for “health keeping” or “therapeutic” intended uses. A common aspect of scientific proof is that for both types of products it has to be obtained through studies involving human beings, generally epidemiological data or clinical studies. This will be a mandatory direction for research on botanical products since it is not more possible effective health use of botanical products in absence of clinical studies. Even if scientific preclinical experiments could seem as secondary in the new scenario, this does not mean that they are not important. Preclinical investigation keeps to be the primary and fundamental proof to address successive research for clinical evidence. For many herbal preparations contained in well-established or traditional herbal medicinal products an adequate safety profile, may be confirmed by their long-term medicinal and/or food use. However, in cases where a safety concern is recognized or suspected, non-clinical investigations may be needed. The documented experience gathered during the long-standing use generally represents the main basis of the pre-clinical assessment both for traditional and well-established herbal medicinal products [24]. However, particular attention should be paid to effects that are difficult or even impossible to detect clinically. In particular, information coming from preclinical toxicological experiments (i.e., genotoxicity, carcinogenicity, and reproductive studies) is indispensable for safety use of botanical products in humans [25]. Genotoxicity studies are designed to detect genetic damage such as gene mutations and chromosomal aberration, which may reflect teratogenic and tumorigenic potential of pharmaceuticals, including herbals [26]. Botanical drug products in the U.S., like other therapeutic agents, are required to provide genotoxicity information prior to marketing approval [27]. Recent data indicate that the European sponsors of botanical products have increasingly recognized the importance of genotoxic data and, in consequence of this, have prioritized their acquisition in drug development programs. On the basis that genotoxicity studies are highly reproducible, and have high statistical power, by purchasing comparably cost-effective data, botanicals companies should be encouraged to realize them as an early goal in their product development [28].

Carcinogenicity studies should be performed for any herbal intended for use as drug for a duration that is continuous for more than 3 months or 6 months intermittently. While for shorter term period use, carcinogenicity information is generally considered not needed. Carcinogenicity studies are generally not needed in cases where there is no suspicion for a carcinogenic potential. Furthermore, the proposed duration of treatment should also be considered [24]. A crucial issue is represented by botanical ingredients containing chemical compounds that are both genotoxic and carcinogenic. Such compounds include, for example, the allylalkoxybenzenes estragole, methyl eugenol, elemicin, tetramethoxyalkylbenzene, safrole, myristicin, and apiole [29]. Unfortunately, in these cases, assessment of the risk to human health is complicated, and an international scientific agreement concerning the best strategy for the risk assessment of genotoxic and carcinogenic compounds is still lacking [30]. 

Reproductive toxicity studies are useful to support the safe use of botanicals; however, these studies are not always necessary. This is the case of botanicals which are designed for postmenopausal symptoms or for benign prostate hyperplasia. The only condition for which there is a cause for concern is for products explicitly indicated in pregnancy [24]. In general, procedures to assess reproductive toxicology should comprise the evaluation of the potential to affect fertility or early embryonic development to implantation, as well as teratology in both a rodent species and a mammalian nonrodent species, and effects on pre- and postnatal development, including maternal function [26]. Another toxicological issue is regarding carcinogenicity information. About the need to clarify toxicological issues, results from postmarketing studies or epidemiological data of adequate power or postmarketing safety studies are always auspicable. 

Because of the complexity and diversity of chemicals present in botanicals, requirements of pharmacokinetic data are almost always limited. Pharmacokinetic findings that sometimes could be useful are those investigating on the inductive or inhibitory effects on P-glycoprotein drug transporters and hepatic P-450 or other drug metabolizing enzyme systems, and those predicting potential herb-drug interactions [31]. Many herbal compounds undergo metabolism *in vivo*, with a major role played by cytochrome P450s enzymes and uridine diphosphate glucuronosyltransferases playing a major role. Some herbal chemicals are substrates of intestinal, hepatic, cerebral and renal P-glycoprotein. Thus, the activities of these drug metabolizing enzymes and drug transporters are determining factors for the *in vivo* bioavailability, disposition, and distribution of herbal chemical substances. Pharmacokinetic studies of botanicals have been mainly focused on a small number of herbal medicines and purified herbal ingredients, including anthocyanins, berberine, catechins, curcumin, hypericin, hyperforin, lutein, and quercetin. For the majority of herbal remedies used in folk medicines, data on their disposition and biological fate in humans are poor or lacking [32]. About the potential herb-drug interactions, it has been suggested to consider it not to be a major issue among botanical safety concerns. The reason is that (a) a minority of herbal preparations, herb-drug interactions seem to be clinically relevant and (b) the inclusion of adequate information on such interactions into the package leaflet could be sufficient for the safe use of the products [33]. The most common botanical-drug interactions that have been described involve herbals like ginkgo biloba (*Ginkgo biloba*), gingseng (*Panax gingseng*), and St. John's wort (*Hypericum perforatum*) [30]. 

Finally, product destination in the market also influences both preclinical and clinical research. From now on, it will become evident that clinical design to study the effects of botanical products has to be a “dress made to measure” according to what the researcher want to demonstrate. In this way, if the development of a botanical product is for medicinal products market, it will be necessary to show beneficial effects in the care of affected subjects (patients). In the case of botanical products for the food supplements market, aim of clinical research has to show that products are able to maintain health state, and in consequence studies will be conducted on healthy subjects. This type of clinical study, apparently easier, puts problems for clinicians normally used to design clinical study aimed to show therapeutic effects in patients. Obviously, if this is not taken in account, the risk is to conduct a study which does not reach the right objective, producing results not suitable for the intended use. On the basis of the different requests of scientific proof for food or medicinal market, also preclinical research should be addressed in the right way. So, if it is thought about a botanical product for food supplements market, research should be built with the objective to show that the product is suitable to keep health and not to care for one or more pathologies. In this case, as an example, there is a more focused experimental model showing that the intake of the product investigated reduces or abolishes the occurrence of a certain pathology, instead that experiments demonstrating that a product cares for an already present pathology. In conclusion, research and development on botanical products is today strongly influenced by the product destination in the market. For this reason, it is necessary that research is addressed and differentiated on the basis of the final market destination. Only in this way adequate safety and efficacy data can be provided for each product category. 
